# ‘I never thought exercise could help improve my sleep’: experiences of people with rheumatoid arthritis on the impact of an 8-week walking-based exercise intervention in improving their sleep

**DOI:** 10.1093/rap/rkae008

**Published:** 2024-01-13

**Authors:** Seán G McKenna, Louise Larkin, Alan Donnelly, Bente Appel Esbensen, Wan Lin Ng, Aqeel Maqsood Anjum, Alexander Fraser, Norelee Kennedy

**Affiliations:** Department of Physiotherapy, Health Service Executive (HSE), University Hospital Limerick Group (UHLG), Limerick, Ireland; Discipline of Physiotherapy, School of Allied Health, University of Limerick, Limerick, Ireland; Health Research Institute (HRI), University of Limerick, Limerick, Ireland; Discipline of Physiotherapy, School of Allied Health, University of Limerick, Limerick, Ireland; Health Research Institute (HRI), University of Limerick, Limerick, Ireland; Health Research Institute (HRI), University of Limerick, Limerick, Ireland; Department of Physical Education and Sport Sciences, University of Limerick, Limerick, Ireland; Copenhagen Center for Arthritis Research, Center for Rheumatology and Spine Diseases, Center for Head and Orthopedics, Rigshospitalet, Denmark; Department of Clinical Medicine, Faculty of Health and Medical Sciences, University of Copenhagen, Copenhagen, Denmark; Department of Rheumatology, Tallaght University Hospital, Dublin, Ireland; Department of Rheumatology, University Hospitals Group Limerick, Limerick, Ireland; Department of Rheumatology, University Hospitals Group Limerick, Limerick, Ireland; Graduate Entry Medical School (GEMS), University of Limerick, Limerick, Ireland; Discipline of Physiotherapy, School of Allied Health, University of Limerick, Limerick, Ireland; Health Research Institute (HRI), University of Limerick, Limerick, Ireland

**Keywords:** inflammatory arthritis, physical activity, physiotherapy, sleep, mental health, qualitative

## Abstract

**Objective:**

The purpose of this study was to explore the experiences of people with RA of participating in an exercise intervention to improve their sleep.

**Methods:**

Using a qualitative descriptive design, semi-structured face-to-face interviews were conducted with 12 people with RA who had completed an 8-week walking-based exercise intervention to improve their total sleep time, sleep quality and sleep disturbance. Data were analysed using thematic analysis.

**Results:**

Four themes were generated: positive impact of exercise on participants’ sleep (‘I really didn’t think any type of exercise would help me sleep better, if I’m honest’); positive experiences of the exercise intervention (‘I learnt so much regarding walking that I didn’t even think about’); clear mental health benefits (‘If you don’t sleep well then it will have a knock-on effect to your mental health’); and achieving empowerment and ownership when exercising (‘I feel empowered now and confident that I’m not doing harm to myself’).

**Conclusion:**

The findings demonstrated that participants had not expected exercise to improve their sleep. Although there is a growing consensus that exercise will benefit sleep and mitigate some disease symptoms, research is severely lacking in people with RA.

Key messages

- Exercise can help people with RA to improve their sleep.
- Further evidence that walking can improve the mental health of people with RA.
- Exercise can help the empowerment and confidence of people with RA when exercising.
- The Borg rating of perceived exertion (RPE) (range 6–20) is a beneficial form of monitoring their exertion.

## Introduction

RA is an autoimmune and inflammatory disease, causing inflammation and pain in the affected parts of the body, usually many joints [1]. There is strong evidence to suggest that for people with RA, increasing their physical activity and exercise can help to improve symptoms and reduce the overall impact of systemic manifestations [2, 3]. Exercise, being a subset of physical activity [4], is a cornerstone of treatment for symptoms among people with RA [5, 6], with current rheumatology guidelines recommending exercise as an integral management of people with RA [7, 8]. Benefits of participation in regular exercise for people with RA include improvements in cardiovascular health, muscle strength and functional ability, reduced pain and fatigue, in addition to improvements in health-related quality of life [9–11].

Sleep is an essential aspect in maintaining the circadian rhythm of the body and health-related quality of life [12]. Poor sleep quality and sleep disturbances are prevalent complaints in people with RA [13, 14], with poor sleep quality and low total sleep time (TST) noted in recent studies [15, 16]. Exercise is known to help improve sleep [17, 18], with some initial trials in people with inflammatory arthritis suggesting that it might help this population [19, 20]. Previous qualitative research on the effects of exercise among people with RA has also examined how traditional exercise is experienced. However, there is a distinct lack of both quantitative and qualitative information regarding exercise and its impact on sleep quality [15, 19, 21].

Previously, we conducted a randomized controlled trial (RCT) examining the feasibility of an 8-week walking-based exercise intervention on TST, sleep quality and sleep disturbance [20, 22]. Data from this pilot trial indicated that a walking-based intervention was both feasible and safe for people with RA who have poor sleep, moderate to severe disability and moderate disease activity, with no adverse events.

The aim of this study was to explore the perceptions of participants of the impact of an 8-week walking-based exercise intervention on TST, sleep quality and sleep disturbance in order to inform a larger trial design. Interviews with participants would add crucial information from them to inform future development of exercise interventions, in addition to exploring the views of participants on the feasibility, barriers and benefits of an exercise intervention in improving sleep.

## Methods

### Study design

A qualitative descriptive study was undertaken to capture the experience of participants of an exercise intervention using face-to-face semi-structured interviews, which was followed by thematic analysis. The consolidated criteria for reporting qualitative research (COREQ) checklist provided guidance during the reporting of this study [23] (see Supplementary Material, available at *Rheumatology Advances in Practice* online).

To ensure that the questions had a valid and meaningful theoretical scaffolding, the questioning route or topic guide for this study was generated based on a literature review of relevant research [24] and experiences from the feasibility study. This topic guide was then refined after discussion with the research team to ensure that the questions, content and structure were suitably open-ended, neutral and sensitive [25].

### Participants and recruitment

Participants were recruited from a pilot RCT investigating the feasibility of an 8-week walking-based intervention to improve sleep quality in people with RA [22]. All participants from the intervention group (*n* = 10) accepted the invitation to participate in face-to-face semi-structured interviews. In addition, participants from the control group (*n* = 10), who were given the opportunity to participate in the intervention post study, were also invited, with two agreeing to participate. Ethical approval for the study was granted by the Research Ethics Committee at the University Hospitals Limerick, Ireland (REC reference 60/17). All procedures performed in studies involving human participants were in accordance with the ethical standards of the University and with the 1964 Declaration of Helsinki and its later amendments or comparable ethical standards.

### Interview guide and data collection

Interview questions were developed from an extensive review of the literature on exercise interventions in people who have RA and of the effects of exercise on sleep in the general population. The questions were subject to discussion within the research team, which consisted of two physiotherapists and a consultant rheumatologist, and were amended and revised based on these discussions. The interview questions covered the participants’ experiences of the exercise intervention, their views on the outcome measures used, and the impact of the intervention on their sleep. The questioning route explored the participants’ experience of the intervention, the outcome measures used, their views regarding the frequency, intensity, time and type of the intervention, in addition to their perceptions regarding exercise and sleep and the benefits of exercise in improving sleep. These were developed into an interview guide (Table 1).

**Table 1.** Interview guide

Topic	Example questions
Experience of exercise intervention	What did you think of the exercise intervention?
	What are your views on the frequency, intensity, time and type, i.e. design of the intervention?
	Did you find the Borg rating of perceived exertion (RPE) 6–20 relevant and useful?
	How did you find the progressions from week to week?
	What did you think of the outcome measures we used? Were they easy to complete? Did they make sense?
	Do you think they picked up on sleep and mood issues that are important to you? Did the questions make sense to you?
	Did they ask about things that were relevant to you?
	How did you find the process of the exercise intervention?
	Is there anything we can do to improve on it?
	Were the reminders useful? How did you find the frequency of the reminders?
	Is there anything else you think we should be measuring as part of improving sleep and mood, with regard to an exercise?
	Is there anything else that you would like to add on the exercise intervention? Or about the design to test the impact of exercise on sleep?
Impression of exercise and sleep	What is your opinion on exercise and sleep? Have you always held that belief?
	Is there anything else that could improve your sleep? Why? Have your beliefs changed over the past number of months?
	Would you go for a nap during the day? Why/why not?
	Is there anything further you would like to add regarding the impact of exercise on sleep?

Face-to-face semi-structured interviews using this interview guide were carried out by the first author, who was known to the participants by being the instructor during the pilot RCT exercise intervention. The interviews were recorded at a university facility, with interviews being undertaken over a 2-week period, immediately after the pilot RCT and over a 1-week period for those in the control group who participated in the exercise intervention post study.

Interviews typically lasted between 30 and 45 min and, before each interview, the first author sought to develop rapport with the participants, in order to provide a relaxed environment. In addition, he reflected on his interview technique and style after the first couple of interviews to enhance those coming after. Interviews were recorded using a digital voice recorder, and during the interviews, the researcher took notes, as needed. At the conclusion of each interview, the first author debriefed the participant on the main content of the interview, and time was permitted for any additional commentary to facilitate the emergence of new, unanticipated information [26].

### Data analysis

Interviews were transcribed verbatim by a professional transcriber. Each participant was offered a copy of their transcript to review, with three taking up the opportunity. The participants were advised to amend the transcript if they saw fit; none of the three amended theirs. The analytical approach for these data was thematic analysis, which was guided by the method of Braun and Clarke [27]. This approach was chosen because it is flexible while also providing a rich, detailed, but complex account of any data set.

We followed the six steps as outlined by Braun and Clarke [27], namely familiarization with the data, generation of initial codes, searching for themes, reviewing themes, defining and naming themes and producing a report. In step 1, all transcripts were transcribed verbatim and transferred into Excel. Errors were checked within each transcript against the tape recording; transcripts were read several times, and initial ideas were noted. The second step involved the generation of preliminary codes. These codes were drawn from key words and phrases related to the research question, separately by two reviewers (first and second authors). To promote rigour, each of these reviewers read and re-read the transcripts, from which they devised the coding list, which was then compared, discussed and amalgamated, and a comprehensive list of codes was finalized. The finalized code list was then applied to all transcripts by the first author. In the third step, through a process of repetitive interpretation, synthesizing and theorizing themes were sought [28]. Similar codes were grouped into subthemes, which were then examined to identify how these might form themes. In step 4, transcripts were then re-read several times, and the selected themes were finalized based on consensus discussion between the first, second and last study authors. Throughout data collection and analysis, widely accepted strategies for ensuring quality in qualitative analysis were maintained, including auditability, fit and transferability [29]. The fifth step included the defining and naming of these themes. Step 6, the final step, involved the selection of quotations from the raw data to best capture themes and subthemes, for which the author could then write up the results for the purpose of an academic paper. The final report was forwarded to the individual participants, who did not suggest any changes. This was important to ensure that the study participants were partners with researchers to help decide what research is done and how it is done [30].

## Results

### Patient characteristics

Twelve face-to-face semi-structured interviews were conducted. All participants were female, with a mean age of 58 (s.d. 7.4) years, a mean RA diagnosis of 9.9 (s.d. 7.4) years and moderate to severe disability [HAQ-disability index: 1.5 (s.d. 0.60]. Summary participant characteristics are provided in Table 2, with selected individual participant characteristics outlined in Table 3.

**Table 2.** Summary participant characteristics (*n* = 12)

Demographic data	*n* (%)
Age, mean ± s.d., years	58 ± 7.4
RA symptoms, mean ± s.d., years	14.1 ± 7.4
RA diagnosed, mean ± s.d., years	9.9 ± 7.0
Gender (male/female)	0/10 (100)
Relationship status	
Single	2 (20)
Married	8 (80)
Working situation	
Full-time employment	1 (10)
Part-time employment	5 (50)
Disability pension	1 (10)
Housewife/househusband	2 (20)
Unemployed	0 0
Retired (owing to age)	1 (10)
Education	
Primary level	1 (10)
Secondary level	3 (30)
Tertiary level	6 (60)
Smoking status	
Now smoking	2 (20)
Used to smoke	5 (50)
Take alcohol	8 (80)
Type of RA medication	
Biological DMARDs	7 (70)
Non-biological DMARDs	2 (20)
Don’t take medication	0 (0)
Don’t know	1 (10)
Sleep medication	
None	5 (50)
Prescribed	3 (30)
Over the counter	2 (20)
BMI, mean ± s.d., kg/m^2^	27 ± 4.1
Maximal O_2_ uptake, mean ± s.d., ml/kg/min	27.4 (5.4)^a^
MVPA, median (IQR), min	152 (93, 211)^a^
HAQ	1.5 ± 0.60
PSQI eligibility score (0/21), mean ± s.d.	13.4 ± 2.5
NYHA classification	Class I = 9 Class II = 1

IQR: interquartile range; MVPA: moderate to vigorous physical activity; NYHA: New York Heart Association; PSQI: Pittsburgh sleep quality index.

a Data available for 8 of 10.

**Table 3.** Selected individual characteristics (*n* = 12)

Participant number	Age (years)	RA symptoms (years)	RA diagnosed (years)	PSQI pre (range 0–21)	PSQI post (range 0–21)	Sleep medication	Working situation	Education
SL01	58	16	14	13	9	None	Housewife	Secondary
SL04	57	16	11	13	7	Prescribed	Part-time	Tertiary
SL05								
SL06	60	6	2	11	5	None	Part-time	Secondary
SL09	51	15	14	11	8	None	Disability	Tertiary
SL11	68	23	14	11	8	Over the counter	Retired	Primary
SL12	62	4	3	11	8	Over the counter	Part-time	Tertiary
SL14	62	15	5	13	5	None	Full-time	Tertiary
SL15	46	8	6	17	7	Prescribed	Part-time	Tertiary
SL17	50	9	4	17	5	Prescribed	Part-time	Tertiary
SL20	68	29	26	17	6	None	Housewife	Secondary
SL22	49	10	6	9	4	None	Full-time	Tertiary

PSQI: Pittsburgh sleep quality index; SL: Sleep study participant.

### Themes

Four themes were identified from the data. These, along with supporting quotations, are presented in the text and in Table 4. The themes identified from the data are as follows: positive impact of exercise on participants’ sleep; positive experiences of the exercise intervention; clear mental health benefits; and achieving empowerment and ownership when exercising.

**Table 4.** Generated themes, with supporting quotations

Theme	Sample quotation (participant number)
Positive impact of exercise on participants’ sleep	‘I really didn’t think any type of exercise would help me sleep better, if I’m honest.’ (SL01)
	‘I never thought exercise would help me sleep better…’ (SL17)
	‘I would have thought I would be full of energy and not be able to sleep, but that didn’t happen, did it?’ (SL20)
	‘they were a great focus … the sleep diary really made me think about my bad habits.’ (SL01)
	‘Yes, good prompt alright. Helped me to focus on my sleep and realize where I could improve….’ (SL20)
	‘…gee, this programme has certainly helped changed my view on it.’ (SL17)
	‘It actually has changed how I look at exercise and sleep now, you know.’ (SL06)
Positive experiences of the exercise intervention	‘I learnt so much regarding walking that I didn’t even think about.’ (SL17)
	‘…really enjoyed it and found it very beneficial for my sleep.’ (SL14)
	‘…had a little bit of muscle ache in my legs, but you told us we might have, so I wasn’t too worried.’ (SL04)
	‘So, the warm-up was great to get me limbered and mobile, but I never really heard of it before, and it really makes such a difference in how I can handle the distance.’ (SL06)
	‘…before, I would go for a walk, I would get stuck in straight away, and my shins would be sore afterwards.’ (SL09)
	‘…the importance of warming up and, you know, what it really made me go a bit further, as I wasn’t as out of breath as usual.’ (SL12)
	‘I’ve really felt the benefit of my upper back and, I dunno, my arms are easier to swing when I’m walking.’ (SL01)
	‘…the stretching was great, so much better than holding the muscle.’ (SL15)
	‘…what I really liked was what I think you called the pacing and that form we used to monitor the intensity was only great.’ (SL01)
	‘I found that really easy to use, so simple isn’t it!’ (SL06)
	‘…the one thing I suppose I hadn’t maybe known about was the upping the heart rate bit.’ (SL09)
	‘…suppose I didn’t really know how to push myself, there was no middle ground.’ (SL17)
	‘…I’m taking away from the course is pacing myself but increasing it now rather than just strolling.’ (SL15)
Clear mental health benefits	‘I was actually very surprised on how much was focused on state of mind, mood.’ (SL06)
	‘I’d never considered it before. But I suppose it’s all linked, isn’t it? If you don’t sleep well then it will have a knock-on effect to your health.’ (SL15)
	‘The depression ones were close to the bone at times.’ (SL17)
	‘There’s always that tendency not to talk about depression and sleep … I’m so glad I signed up for it all as I now feel able to discuss mental health with the family.’ (SL09)
	‘We don’t talk about it much in this country … so we shouldn’t be embarrassed, and I think, please, please keep them in!’ (SL01)
	‘I honestly believe it was around my diagnosis that I suffered from depression, but sure, there was no mention of it. Now, though, it should be discussed and not shied away from!’ (SL15)
Achieving empowerment and ownership when exercising	‘It took me a while, but writing things down of what I’d done has helped me take ownership rather than putting it on someone else.’ (SL15)
	‘I feel empowered now and confident that I’m not doing harm to myself.’ (SL17)
	‘Because I’m able to pace myself better now, I feel that I can really say that my daily exercise is my own.’ (SL20)
	‘…had never been to one before…. It’s a pity coz we’d learn a lot from ye!’ (SL20)
	‘Being coached by a physiotherapist might have made a difference to me continuing with it.’ (SL06)
	‘Never been given the opportunity to visit a physio before….’ (SL20)
	‘My sister laughed at the start when I told her I was participating in a walking programme to help me sleep better. Now I’m laughing!’ (SL09)
	‘I’ve a couple of friends who I used to walk with but had to stop coz it was too fast, and I felt they weren’t supporting me.’ (SL06)

#### Positive impact of exercise on participants’ sleep

Participants described their experiences of exercise and its impact on sleep. This theme encapsulated the participants’ understanding of exercise and any impact it might have had on sleep. Most participants were clearly surprised that exercise could improve sleep. This was described as follows:

‘I really didn’t think any type of exercise would help me sleep better, if I’m honest.’ (SL01)

‘I never thought exercise could help improve my sleep….’ (SL17)

As part of the pilot RCT, participants completed the National Sleep Foundation’s sleep diary in order to assess their sleeping pattern. Some participants discussed the importance of this in monitoring their sleep and exercise levels:

‘they were a great focus … the sleep diary really made me think about my bad habits.’ (SL01)

In addition, several participants commented on how the outcome measures and exercise logs used helped them to be more aware of improvements in their sleep and exercise levels. Several participants illustrated this:

‘…it was only when I was completing that barriers scale that I realized I’d changed my opinion on exercise helping me sleep.’ (SL12)

Participants described how their involvement in the exercise intervention changed their perception regarding exercise improving sleep:

‘…gee, this programme has certainly helped changed my view on it.’ (SL17)

#### Positive experiences of the exercise intervention

In this theme, participants discussed their experiences of participating in the exercise intervention. Study satisfaction was high, with all participants reporting that they had been sufficiently informed about the study before giving consent, and all were satisfied/very satisfied with being in the study.

‘…really enjoyed it and found it very beneficial for my sleep.’ (SL14)

Participants spoke about the benefit of warming up and dynamic stretching. Several participants were surprised at the benefit of warming up:

‘…the importance of warming up and, you know, what it really made me go a bit further as I wasn’t as out of breath as usual.’ (SL12)

In addition, the concept of dynamic stretching, which was new to several participants, was found to be very beneficial:

‘I’ve really felt the benefit of my upper back and, I dunno, my arms are easier to swing when I’m walking’ (SL01)

The exercise programme was devised using incremental targets for daily walks, and participants were advised to monitor and progress their exercise intensity using the Borg rating of perceived exertion (RPE) scale (range 6–20) [31]. Participants found this form of monitoring very beneficial:

‘…what I really liked was what I think you called the pacing and that form we used to monitor the intensity was only great.’ (SL01)

In addition, several participants (*n* = 5) mentioned the realization of the importance of increasing their heart rate, which they weren’t aware of previously:

‘…suppose I didn’t really know how to push myself, there was no middle ground.’ (SL17)

#### Clear mental health benefits

This theme encapsulated the participants’ surprise and initial unease at completing several depression and anxiety outcome measures, as part of their participation in the exercise intervention. Comments relating to their initial completion included:

‘I was actually very surprised on how much was focused on state of mind, mood.’ (SL06)

‘I’d never considered it before. But I suppose it’s all linked, isn’t it? If you don’t sleep well then it will have a knock-on effect to your health.’ (SL15)

However, after the intervention, participants felt they had been given the opportunity to allow them to discuss the issue of mental health:

‘There’s always that tendency not to talk about depression and sleep … I’m so glad I signed up for it all, as I now feel able to discuss mental health with the family.’ (SL09)

‘We don’t talk about it much in this country … so we shouldn’t be embarrassed, and I think, please, please keep them in!’ (SL01)

#### Achieving empowerment and ownership when exercising

The fourth theme generated from the data describes how participants have achieved an element of empowerment and confidence when exercising. Participants discussed the importance of having a goal or plan when exercising, which also straddled a few other themes.

‘It took me a while, but writing things down of what I’d done has helped me take ownership rather than putting it on someone else’ (SL15)

‘I feel empowered now and confident that I’m not doing harm to myself.’ (SL17)

Interestingly, most participants (*n* = 7) had never been seen by a physiotherapist to help with their RA.

‘…had never been to one before…. It’s a pity coz we’d learn a lot from ye!’ (SL20)

‘Being coached by a physiotherapist might have made a difference to me continuing with it.’ (SL06)

Participants who had set goals and who had achieved them described how support from family and friends could be both a benefit and a barrier.

‘I’ve a couple of friends who I used to walk with but had to stop coz it was too fast, and I felt they weren’t supporting me.’ (SL06)

At present, these sources of support mentioned were primarily family and friends and not professionals. Participants spoke about these sources of support and the importance of identifying the need for and sourcing further information from a physiotherapist, not only in managing their RA, but also information regarding what to do to improve their sleep.

## Discussion

This study aimed to evaluate qualitatively the experiences of participants in an 8-week walking-based intervention and the impact of this programme on TST, sleep quality and sleep disturbance. Overall, participants expressed a positive attitude to the exercise intervention allowing them a sense of ownership when exercising, with clear mental health benefits. These effects are consistent with meta-analytical findings for improved mental health after exercise in both healthy and chronically ill populations, including those with RA [32, 33]. Although people with RA are less active and engage in lower levels of exercise compared with their healthy counterparts [34], participants in this study occasionally engaged in exercise to help manage their symptoms. The results from this study highlight that participants were surprised that exercise could help to improve sleep. Moreover, interviewees described the mental health benefits experienced after exercise to improve sleep, thus extending evidence on the perceived beneficial impact of participation in exercise, acknowledged by this population [35].

In a variety of conditions, such as cardiovascular disease (CVD), type 2 diabetes, depression and some cancers, physical activity and exercise have been shown to have a positive impact on sleep [21, 36]. A significant amount of research has been aimed at understanding the physiology of sleep and the interrelationship between sleep and exercise [37]. Although there is significant research surrounding sleep and exercise as they affect one another in multiple, diverse populations, the specific physiological factors by which the two interact remain undefined [37].

Although there is a growing consensus that exercise will benefit sleep for those experiencing a chronic health disorder, research is lacking, in particular for those with a rheumatic condition [18, 37]. Because of the multifactorial nature of RA and to help mitigate some of its symptoms, engaging in exercise has been widely researched and advised, with our study adding to the existing understanding of the impact of exercise on people with RA to help improve their sleep [38]. Given that exercise prescription is a core skill for some health-care professionals (e.g. physiotherapists) [39], they play an important role in educating people with RA on the benefit of increasing their exercise levels [40, 41] in improving their TST and sleep quality. Given that a recent systematic review has provided further evidence that being physically active is an important contributor to symptom management in people with RA [42], it is essential to explore negative beliefs regarding the impact of exercise on sleep among health-care professionals when seeking to promote exercise.

This study provided some compelling comments regarding the mental health of participants. Participants seemed to be uneasy in completing depression and/or anxiety outcome measures. However, they acknowledged being more open regarding discussion of these topics and appreciated the opportunity to be open after the intervention. It has been reported that the degree of depression in people with RA is a preceding sign of physical disability that can appear later in life [43]; therefore, any psychiatric sign could be as much of a prediction as any biological sign in the prognosis of the disease. With meta-regression analytical results [32] indicating the significant effect of exercise on reducing the impact of these psychiatric co-morbidities, health-care professionals should be confident in prescribing exercise to people with RA who have depression and anxiety, because it significantly reduces their symptoms.

Participants expressed a need for support and education when starting an exercise programme and spoke about the link between support and its resultant empowerment and ownership, with their comments reflecting health behaviour change literature. Both the social cognitive theory [44] and health action process approach [45] support this link, with the latter suggesting that a positive outcome expectation (e.g. if I exercise, my sleep might improve) is chiefly important in the motivational phase of changing behaviour. Aiming to improve TST and sleep quality through increasing exercise might be a health-promotion strategy that is feasible and safe for this population [46–48].

### Study strengths and limitations

A potential limitation relates to the recruitment of the participants, which might have resulted in volunteer bias. The fact that these participants were willing to engage in an exercise intervention study might indicate that these participants displayed similar views and experiences relating to their sleep and were at least willing to consider an exercise intervention to improve sleep. However, as with all qualitative research, the aim is to improve the understanding and not generalizability; therefore, the richness of the participants’ replies is essential.

The same person interviewed all the participants, ensuring homogeneity throughout the interviews; however, this person was also involved in the intervention, which introduces a certain amount of bias.

All those in the intervention group participated, in addition to a number from the control group, who provided further insights. This was particularly interesting because some of those from the exercise study who agreed to participate did not experience an improvement in their sleep quality. Nevertheless, they were willing to share their experiences because they had perceived participation in the pilot RCT to be a positive experience.

The interview route was designed through a process of consultation amongst the study authors, with interviews undertaken by the first author. He probed other issues that arose and encouraged people to discuss whatever they felt pertinent.

Finally, the participants in this study were all female, independently mobile and able to be active and therefore might not be representative of those with greater mobility limitations and with a variety of activity levels.

## Conclusion

The themes emerging from this study increase our insight into the experiences of patients participating in an 8-week exercise intervention to improve sleep. The results demonstrated that participants were clearly surprised that exercise could improve their sleep quality and found it beneficial and safe.

The positive improvements in sleep outcomes reported by the participants provide preliminary evidence for the effect of a physiotherapist-led walking intervention on sleep in a sample of people with RA. Participants expressed positive comments in relationship to the intervention, including overall enjoyment of the exercise programme. These findings should inform the design for a future larger RCT of a walking-based exercise intervention for people with RA to improve TST, sleep quality and sleep disturbances.

## Supplementary Material

rkae008_Supplementary_Data

## Data Availability

Deidentified data obtained, used and/or analysed during the current study are available from the corresponding author upon reasonable request.
